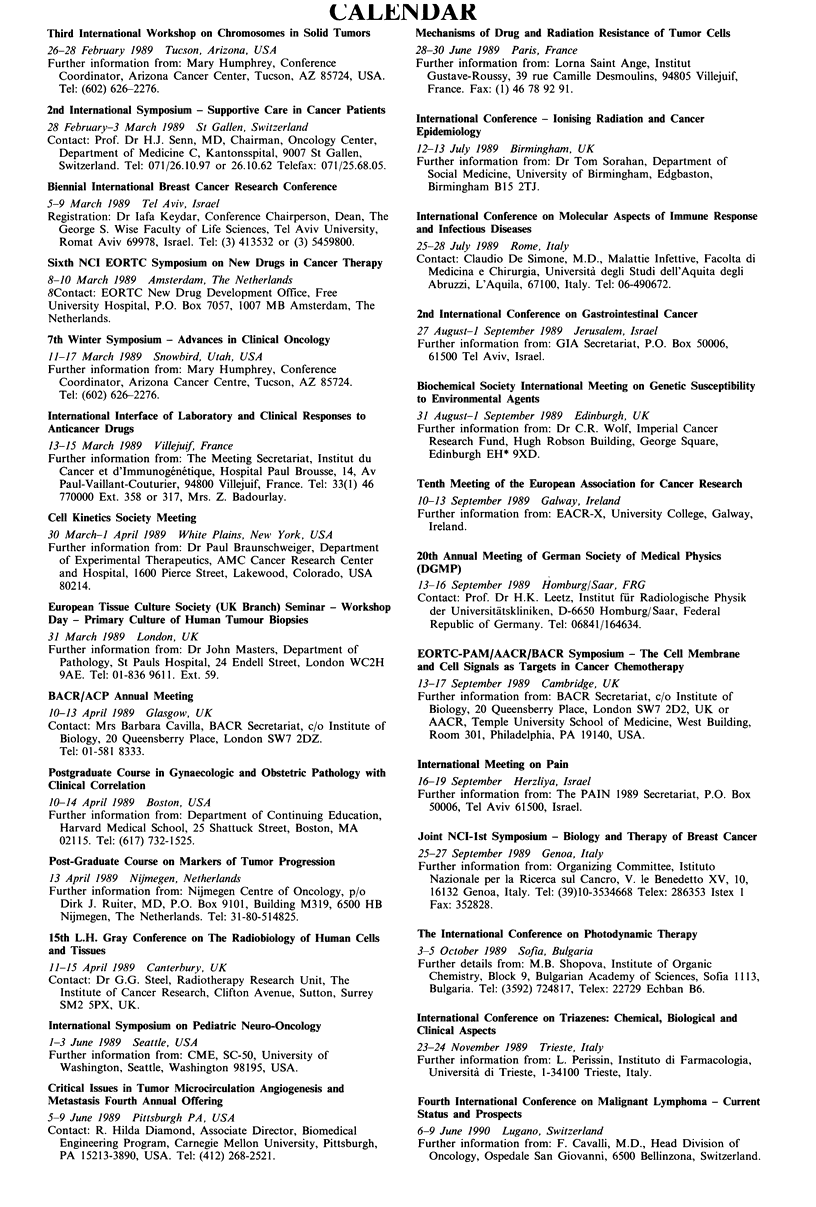# Calendar

**Published:** 1989-02

**Authors:** 


### CALENDAR

**Third International Workshop on Chromosomes in Solid Tumors**

*26–28 February 1989 Tucson, Arizona, USA*

Further information from: Mary Humphrey, Conference Coordinator, Arizona Cancer Center, Tucson, AZ 85724, USA. Tel: (602) 626–2276.

**2nd International Symposium – Supportive Care in Cancer Patients**

*28 February–3 March 1989 St Gallen, Switzerland*

Contact: Prof. Dr H.J. Senn, MD, Chairman, Oncology Center, Department of Medicine C, Kantonsspital, 9007 St Gallen, Switzerland. Tel: 071/26.10.97 or 26.10.62 Telefax: 071/25.68.05.

**Biennial International Breast Cancer Research Conference**

*5–9 March 1989 Tel Aviv, Israel*

Registration: Dr Iafa Keydar, Conference Chairperson, Dean, The George S. Wise Faculty of Life Sciences, Tel Aviv University, Romat Aviv 69978, Israel. Tel: (3) 413532 or (3) 5459800.

**Sixth NCI EORTC Symposium on New Drugs in Cancer Therapy**

*8–10 March 1989 Amsterdam, The Netherlands*

*8*Contact: EORTC New Drug Development Office, Free University Hospital, P.O. Box 7057, 1007 MB Amsterdam, The Netherlands.

**7th Winter Symposium – Advances in Clinical Oncology**

*11–17 March 1989 Snowbird, Utah, USA*

Further information from: Mary Humphrey, Conference Coordinator, Arizona Cancer Centre, Tucson, AZ 85724. Tel: (602) 626–2276.

**International Interface of Laboratory and Clinical Responses to Anticancer Drugs**

*13–15 March 1989 Villejuif, France*

Further information from: The Meeting Secretariat, Institut du Cancer et d'Immunogénétique, Hospital Paul Brousse, 14, Av Paul-Vaillant-Couturier, 94800 Villejuif, France. Tel: 33(1) 46 770000 Ext. 358 or 317, Mrs. Z. Badourlay.

**Cell Kinetics Society Meeting**

*30 March–1 April 1989 White Plains, New York, USA*

Further information from: Dr Paul Braunschweiger, Department of Experimental Therapeutics, AMC Cancer Research Center and Hospital, 1600 Pierce Street, Lakewood, Colorado, USA 80214.

**European Tissue Culture Society (UK Branch) Seminar – Workshop Day – Primary Culture of Human Tumour Biopsies**

*31 March 1989 London, UK*

Further information from: Dr John Masters, Department of Pathology, St Pauls Hospital, 24 Endell Street, London WC2H 9AE. Tel: 01-836 9611. Ext. 59.

**BACR/ACP Annual Meeting**

*10–13 April 1989 Glasgow, UK*

Contact: Mrs Barbara Cavilla, BACR Secretariat, c/o Institute of Biology, 20 Queensberry Place, London SW7 2DZ. Tel: 01-581 8333.

**Postgraduate Course in Gynaecologic and Obstetric Pathology with Clinical Correlation**

*10–14 April 1989 Boston, USA*

Further information from: Department of Continuing Education, Harvard Medical School, 25 Shattuck Street, Boston, MA 02115. Tel: (617) 732-1525.

**Post-Graduate Course on Markers of Tumor Progression**

*13 April 1989 Nijmegen, Netherlands*

Further information from: Nijmegen Centre of Oncology, p/o Dirk J. Ruiter, MD, P.O. Box 9101, Building M319, 6500 HB Nijmegen, The Netherlands. Tel: 31-80-514825.

**15th L.H. Gray Conference on The Radiobiology of Human Cells and Tissues**

*11–15 April 1989 Canterbury, UK*

Contact: Dr G.G. Steel, Radiotherapy Research Unit, The Institute of Cancer Research, Clifton Avenue, Sutton, Surrey SM2 5PX, UK.

**International Symposium on Pediatric Neuro-Oncology**

*1–3 June 1989 Seattle, USA*

Further information from: CME, SC-50, University of Washington, Seattle, Washington 98195, USA.

**Critical Issues in Tumor Microcirculation Angiogenesis and Metastasis Fourth Annual Offering**

*5–9 June 1989 Pittsburgh PA, USA*

Contact: R. Hilda Diamond, Associate Director, Biomedical Engineering Program, Carnegie Mellon University, Pittsburgh, PA 15213-3890, USA. Tel: (412) 268-2521.

**Mechanisms of Drug and Radiation Resistance of Tumor Cells**

*28–30 June 1989 Paris, France*

Further information from: Lorna Saint Ange, Institut Gustave-Roussy, 39 rue Camille Desmoulins, 94805 Villejuif, France. Fax: (1) 46 78 92 91.

**International Conference – Ionising Radiation and Cancer Epidemiology**

*12–13 July 1989 Birmingham, UK*

Further information from: Dr Tom Sorahan, Department of Social Medicine, University of Birmingham, Edgbaston, Birmingham B15 2TJ.

**International Conference on Molecular Aspects of Immune Response and Infectious Diseases**

*25–28 July 1989 Rome, Italy*

Contact: Claudio De Simone, M.D., Malattie Infettive, Facolta di Medicina e Chirurgia, Università degli Studi dell'Aquita degli Abruzzi, L'Aquila, 67100, Italy. Tel: 06-490672.

**2nd International Conference on Gastrointestinal Cancer**

*27 August–1 September 1989 Jerusalem, Israel*

Further information from: GIA Secretariat, P.O. Box 50006, 61500 Tel Aviv, Israel.

**Biochemical Society International Meeting on Genetic Susceptibility to Environmental Agents**

*31 August–1 September 1989 Edinburgh, UK*

Further information from: Dr C.R. Wolf, Imperial Cancer Research Fund, Hugh Robson Building, George Square, Edinburgh EH* 9XD.

**Tenth Meeting of the European Association for Cancer Research**

*10–13 September 1989 Galway, Ireland*

Further information from: EACR-X, University College, Galway, Ireland.

**20th Annual Meeting of German Society of Medical Physics (DGMP)**

*13–16 September 1989 Homburg/Saar, FRG*

Contact: Prof. Dr H.K. Leetz, Institut für Radiologische Physik der Universitätskliniken, D-6650 Homburg/Saar, Federal Republic of Germany. Tel: 06841/164634.

**EORTC-PAM/AACR/BACR Symposium – The Cell Membrane and Cell Signals as Targets in Cancer Chemotherapy**

*13–17 September 1989 Cambridge, UK*

Further information from: BACR Secretariat, c/o Institute of Biology, 20 Queensberry Place, London SW7 2D2, UK or AACR, Temple University School of Medicine, West Building, Room 301, Philadelphia, PA 19140, USA.

**International Meeting on Pain**

*16–19 September Herzliya, Israel*

Further information from: The PAIN 1989 Secretariat, P.O. Box 50006, Tel Aviv 61500, Israel.

**Joint NCI-1st Symposium – Biology and Therapy of Breast Cancer**

*25–27 September 1989 Genoa, Italy*

Further information from: Organizing Committee, Istituto Nazionale per la Ricerca sul Cancro, V. le Benedetto XV, 10, 16132 Genoa, Italy. Tel: (39)10-3534668 Telex: 286353 Istex 1 Fax: 352828.

**The International Conference on Photodynamic Therapy**

*3–5 October 1989 Sofia, Bulgaria*

Further details from: M.B. Shopova, Institute of Organic Chemistry, Block 9, Bulgarian Academy of Sciences, Sofia 1113, Bulgaria. Tel: (3592) 724817, Telex: 22729 Echban B6.

**International Conference on Triazenes: Chemical, Biological and Clinical Aspects**

*23–24 November 1989 Trieste, Italy*

Further information from: L. Perissin, Instituto di Farmacologia, Università di Trieste, 1-34100 Trieste, Italy.

**Fourth International Conference on Malignant Lymphoma – Current Status and Prospects**

*6–9 June 1990 Lugano, Switzerland*

Further information from: F. Cavalli, M.D., Head Division of Oncology, Ospedale San Giovanni, 6500 Bellinzona, Switzerland.